# Heroin seeking becomes dependent on dorsal striatal dopaminergic mechanisms and can be decreased by N‐acetylcysteine

**DOI:** 10.1111/ejn.13894

**Published:** 2018-03-30

**Authors:** Ritchy Hodebourg, Jennifer E. Murray, Maxime Fouyssac, Mickaël Puaud, Barry J. Everitt, David Belin

**Affiliations:** ^1^ Department of Pharmacology University of Montreal Montreal QC Canada; ^2^ Department of Psychology University of Guelph Guelph ON Canada; ^3^ Department of Psychology University of Cambridge Cambridge CB2 3EB UK; ^4^ Present address: Department of Psychology University of Guelph Guelph ON Canada

**Keywords:** astrocytes, dopamine, glutamate, habits, heroin

## Abstract

The alarming increase in heroin overdoses in the USA is a reminder of the need for efficacious and novel treatments for opiate addiction. This may reflect the relatively poor understanding of the neural basis of heroin, as compared to cocaine, seeking behaviour. While cocaine reinforcement depends on the mesolimbic system, well‐established cocaine seeking is dependent on dorsolateral striatum (aDLS) dopamine‐dependent mechanisms which are disrupted by N‐acetylcysteine, through normalisation of corticostriatal glutamate homeostasis. However, it is unknown whether a functional recruitment of aDLS dopamine‐dependent control over instrumental responding also occurs for heroin seeking, even though heroin reinforcement does not depend on the mesolimbic dopamine system. Lister Hooded rats acquired heroin self‐administration and were subsequently trained to seek heroin daily over prolonged periods of time under the control of drug‐paired cues, as measured under a second‐order schedule of reinforcement. At different stages of training, that is, early on and when heroin seeking behaviour was well established, we measured the sensitivity of drug‐seeking responses to either bilateral aDLS infusions of the dopamine receptor antagonist α‐flupenthixol (5, 10 and 15 μg/side) or systemic administration of N‐acetylcysteine (30, 60 and 90 mg/kg). The results demonstrate that control over heroin seeking behaviour devolves to aDLS dopamine‐dependent mechanisms after extended training. Further aDLS‐dependent well‐established, cue‐controlled heroin seeking was disrupted by N‐acetylcysteine. Comparison with previous data on cocaine suggests that the development of drug seeking habits and the alteration of corticostriatal glutamate homeostasis, which is restored by N‐acetylcysteine, are quantitatively similar between heroin and cocaine.

## Introduction

The opiate epidemic in the USA (Unger, 2017) and the estimated 69 000 individuals dying from opioid overdose each year worldwide (World Health Organisation, 2017) are a reminder that heroin addiction is still a major health burden. Yet, beyond substitution and associated harm reduction strategies that have relatively limited efficacy, there are no treatments that help decrease heroin seeking behaviour (Best *et al*., 2008).

The neurobiological underpinnings of heroin addiction, which cannot be understood only in terms of reinforcement, tolerance or withdrawal (American Psychiatric Association, 2013), have been relatively under‐investigated compared to stimulants such as cocaine (for review, see Belin *et al*., 2013; Everitt & Robbins, 2016; Everitt *et al*., 2017), and the neural basis of heroin seeking behaviour in particular is poorly understood.

Heroin and cocaine ultimately increase extracellular dopamine concentration in the nucleus accumbens (Di Chiara & Imperato, 1988) and trigger adaptations within striatal dopaminergic systems (Volkow *et al*., 2009). However, their molecular targets differ (Badiani *et al*., 2011) so that the reinforcing properties of heroin are much less reliant on ventral striatal dopaminergic mechanisms than those of cocaine (Ettenberg *et al*., 1982; Pettit *et al*., 1984). Additionally, heroin and cocaine trigger different neurobiological adaptations within corticostriatal circuits (for review, see Badiani *et al*., 2011), suggesting the long‐lasting behavioural effects of these two drugs may eventually depend on different neural and psychological processes. Beyond these differences, self‐administered cocaine and heroin result in similar alterations in glutamate homeostasis within the striatum and glutamatergic mechanisms in the nucleus accumbens core (AcbC) that are implicated in the reinstatement of extinguished instrumental responses for both drugs (Kalivas & McFarland, 2003; LaLumiere & Kalivas, 2008). Additionally, restoration of glutamate homeostasis in the AcbC by the cysteine pro‐drug N‐acetylcysteine (NAC) similarly prevents the reinstatement of instrumental responding for cocaine and heroin following extinction after a limited self‐administration history (Zhou & Kalivas, 2008; Reichel *et al*., 2011). Thereby, these data suggest that alterations in glutamatergic homeostasis in the AcbC that are remediated by NAC may represent a common neural adaptation to cocaine and heroin exposure.

Recent evidence from research on cocaine has revealed both in humans and rats that over the course of exposure to the drug, the locus of control over behaviour shifts from the ventral striatum to dopamine‐dependent mechanisms in the anterior dorsal striatum (aDLS). Thus, in humans addicted to cocaine or using cocaine recreationally, presentation of drug‐paired conditioned stimuli (CSs) triggers an increase in dopamine release in the dorsal striatum (Volkow *et al*., 2006; Cox *et al*., 2017). In rats trained to seek cocaine over prolonged periods of time under the influence of conditioned stimuli, the control over drug seeking progressively devolves to dopamine‐dependent aDLS mechanisms (Vanderschuren *et al*., 2005; Belin & Everitt, 2008; Murray *et al*., 2012a, 2015), which also underpin compulsive drug seeking (Jonkman *et al*., 2012).

However, it is not known whether heroin seeking behaviour also becomes progressively controlled by aDLS dopamine‐dependent mechanisms, thereby limiting our understanding of the neural and associated psychological basis of addiction to opiates, which may differ from those hitherto identified for stimulants. N‐acetylcysteine, which also impairs cocaine‐induced DLS‐dependent habitual control over instrumental responding for natural reinforcers (Corbit *et al*., 2014), decreases aDLS dopamine‐dependent cocaine seeking habits (Murray *et al*., 2012b) and facilitates restoration of control over cocaine intake (Ducret *et al*., 2016). Therefore, NAC may have therapeutic utility to decrease maladaptive heroin seeking behaviour.

We therefore tested the hypothesis that aDLS dopamine‐dependent control over cue‐controlled heroin seeking behaviour is progressively recruited after extended training. We further investigated whether N‐acetylcysteine influenced early and well‐established cue‐controlled heroin seeking behaviour. Rats were trained to seek heroin under a second‐order schedule of reinforcement that facilitates the emergence of cue‐controlled drug seeking habits (Belin & Everitt, 2010). At early and late stages of training, we measured the sensitivity of instrumental seeking responses to bilateral intra‐aDLS infusions of the dopamine receptor antagonist α‐flupenthixol (Murray *et al*., 2013) and systemic administration of NAC. In order better to characterise the similarities between the neural and pharmacological mechanisms of the functional recruitment of aDLS control over heroin seeking when it becomes habitual, we further compared the effects of these manipulations to those we have previously reported for cocaine (Murray *et al*., 2012a,b).

## Materials and methods

### Subjects

Male Lister Hooded rats (*n* = 23, Charles River, Kent, UK) were habituated to the colony for 1 week in a 12‐h reverse light : dark cycle (lights off at 07:00 am). Following recovery from surgery and throughout the experiment, rats were fed 20 g chow per day given within 2 h of concluding each daily session. Water was freely available in the home cage. Experiments were performed during the dark phase, 5–7 days per week, and were conducted in accordance with the United Kingdom 1986 Animals (Scientific Procedures) Act, Project Licence 80/2234 as well as the French and European Directives concerning the use of laboratory animals (Decret 87‐848, 19 October 1987 and 2010/63/EU, respectively) following ethical review by the University of Cambridge Animal Welfare and Ethical Review Body (AWERB).

### Drugs

Heroin hydrochloride (Macfarlan‐Smith, Edinburgh, UK or Cooper, France) was dissolved in sterile 0.9% saline. α‐Flupenthixol and *N*‐acetylcysteine (both from Sigma Aldrich, Poole, UK) were dissolved in double‐distilled water and the pH adjusted to 7.2 for the latter (Murray *et al*., 2012b, 2013; Ducret *et al*., 2016). Drug doses are reported in the salt form.

### Surgery

All rats were anaesthetised with an intraperitoneal injection of ketamine hydrochloride (100 mg/kg; Ketaset; Fort Dodge Animal Health Ltd, Southampton, UK) and xylazine (12 mg/kg; Rompun; Bayer, Wuppertal, Germany). They were then implanted with an intravenous catheter (CamCaths, Ely, UK), as previously described (Vanhille *et al*., 2015).

Rats (*n* = 9) were then implanted bilaterally with 22‐gauge guide cannulae (Plastics One, Roanoke, VA, USA) positioned to lie 2 mm above the anterior dorsolateral striatum (AP +1.2, ML ±3, DV −3; Belin & Everitt, 2008); infusion target AP and ML coordinates were measured from bregma, DV coordinates from the skull surface, with the incisor bar set at −3.3 mm, as previously described (Murray *et al*., 2015). From the day before to 7 days after surgery, rats were weighed and treated subcutaneously with 10 mg/kg of the antibiotic Baytril (Bayer). NRats were placed in a temperature controlled recovery cabinet for 2 h following surgery and were subsequently closely monitored in their home cage for 3 days. They all recovered their presurgery body weight within 24 h and did not display clinical signs of suffering or distress, thereby precluding any additional other postoperative care. Catheters were flushed daily throughout the experiment with 0.2–0.4 mL of sterile saline mixed with heparin (20 U/mL; Wockhardt UK Ltd, Wrexham, UK) to maintain patency.

### Pharmacological challenges

Intra‐aDLS infusions of α‐flupenthixol (0.5 μL/side) were made bilaterally over 90 s using a syringe pump (Harvard Apparatus, Holliston, MA, USA) via 28‐gauge steel hypodermic injectors (Plastics One) lowered to the injection sites 2 mm ventral to the end of the guide cannulae (i.e., DLS, −5 mm). Infusions were followed by a 60‐s period to allow diffusion of the infused drug or vehicle before injectors were removed and obturators were replaced. N‐Acetylcysteine (1 mL/kg) was delivered intraperitoneally (IP) at the doses of 30, 60 and 90 mg/kg (Murray *et al*., 2012b). Test sessions began 5 min after intrastriatal infusions (Belin & Everitt, 2008; Murray *et al*., 2012a) and 3 h after intraperitoneal injections (Baker *et al*., 2003; Murray *et al*., 2012b). Test drugs were administered according to a counterbalanced, Latin‐square design.

### Apparatus

Experiments were conducted using 24 standard operant conditioning chambers equipped with two levers as described previously (Murray *et al*., 2012b). Briefly, conditioning chambers (29.5 × 32.5 ×23.5 cm; Med Associates, St. Albans, VT, USA) were each housed in a sound‐ and light‐attenuating cubicle fitted with a ventilation fan. Chambers were equipped with 4‐cm wide retractable levers 8 cm above the grid floor and 12 cm apart. Above each lever was a white cue light (2.5 W, 24 V), and at the top of the opposite wall was a white house light (2.5 W, 24 V). Sidewalls were aluminium; the ceiling, front and back walls were clear polycarbonate. A spring leash was attached to a swivel that connected to a balanced metal arm secured outside of the chamber. Tygon tubing extended from a 10‐mL syringe mounted on a syringe pump (Semat Technical, Herts, UK) located outside each cubical to the swivel and from the swivel, through the leash, to attach to the catheter. Personal computers with Whisker software (Cardinal & Aitken, www.whiskercontrol.com) controlled infusions and light presentations and recorded lever presses.

### Procedure

#### Heroin self‐administration

Following recovery from surgery, all rats were trained to self‐administer heroin (40 μg/infusion; 100 μL/5 s) under a fixed ratio 1 (FR1) schedule of reinforcement. Under this schedule, one active lever press resulted in drug infusion, initiated concurrently with a 20‐s time out that included onset of the cue light positioned above the active lever (conditioned stimulus; CS), offset of the house light and retraction of both levers. Inactive lever pressing was recorded but had no scheduled consequence. Active and inactive lever assignment was counterbalanced, and a maximum of 30 infusions was available for this stage.

Following 10 training sessions, the dose‐dependent effects of both DLS dopamine receptor blockade or systemic administration of NAC on early stage heroin seeking were tested. Thus, following bilateral infusions of α‐flupenthixol into the aDLS (Murray *et al*., 2013) or IP injection of NAC (Murray *et al*., 2012b), rats were challenged in 15‐min test sessions [FI15(FR1:S)] during which every active lever press resulted in a 1‐s light CS presentation, and heroin was only delivered on the first lever press after the 15‐min interval had elapsed (Murray *et al*., 2012a,b, 2013). Thus, the early performance tests were conducted before and were thus unaffected by, self‐administration of heroin. Test sessions were followed by a standard FR1 training session (i.e., up to 30 infusions of heroin in a 2‐h session), and rats had a training session between test days to confirm stable heroin self‐administration baselines.

Following these early stage tests, the daily schedule of reinforcement was changed to fixed intervals, increasing across daily training sessions from 1 min (fixed interval 1‐min, FI2) to FI2, FI4, FI8, FI10 and eventually FI15 min. After three sessions on FI15, the FI15(FR10:S) second‐order schedule of reinforcement was instituted in which completion of each FR10 responses resulted in a 1‐s CS light presentation; heroin was delivered on completion of the first 10 lever presses once each 15‐min fixed interval had elapsed. Rats were trained to respond over 2‐h sessions or for five heroin infusions. Thus, at this final stage rats were trained to seek heroin under the control of heroin‐paired cues for 15 daily sessions after which the sensitivity of well‐established, cue‐controlled heroin seeking behaviour to DLS dopamine receptor blockade or treatment with NAC was assessed. Thus, during these late stage training tests the influence of intra‐aDLS infusions of α‐flupenthixol or IP injection of NAC on heroin seeking behaviour was assessed when it has become well established and under the control of drug‐paired cues (Belin *et al*., 2013).

**Figure 1:**
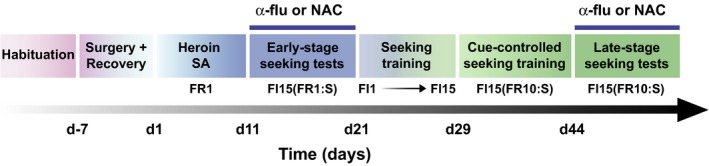
Experimental timeline. Following 1 week of habituation to the colony, Lister Hooded rats underwent either both intravenous catheter implantation and bilateral aDLS cannulations (α‐flupenthixol experiment, α‐flu, *n* = 9) or only the former (N‐acetylcysteine experiment, NAC,* n* = 14) a week before beginning training. Rats acquired heroin self‐administration under a fixed ratio 1 schedule of reinforcement (FR1) under which they were maintained for 10 consecutive daily sessions which were followed by the early stage seeking tests (Murray *et al*., 2012b, 2013, 2015). During the early stage seeking tests, rats were challenged to seek heroin over 15 min with CS presented contingently upon each lever press. From days 21 to 28, the response requirement was increased across sessions to a FI15 (FR10:S) second‐order schedule of reinforcement for heroin. Rats were trained daily to seek heroin under the control of drug‐paired cues for 15 sessions (from days 29 to 43) following which the late stage seeking performance testing began (on day 44). d, day; FI, fixed interval; FR, fixed ratio.

A schematic of the full training and testing timeline is provided in Fig. 1.

### Histology

Rats were euthanised with an overdose of sodium pentobarbital (300 mg; Dolethal; Vétoquinol UK Ltd, Buckingham, UK), and those with intracranial cannulae were perfused transcardially with isotonic saline followed by 10% neutral buffered formalin. Brains were removed and transferred to a 20% sucrose solution in 0.01 m PBS for approximately 24 h before sectioning at 60 μm on a cryostat. Every third section was mounted on a glass slide and stained with cresyl violet. Cannulae placements were verified using a light microscope. Histological assessment was performed blind to experimental results.

### Data and statistical analyses

Data are represented as means ± SEM and were analysed using statistica 10 (Statsoft, Palo Alto, USA). Lever presses during heroin seeking tests were analysed using two‐way analyses of variance (anovas) with lever (active and inactive) and dose (0, 5, 10 and 15 μg/side α‐flupenthixol or 0, 30, 60, 90 mg/kg NAC) as within‐subject factors. Assumptions for normal distribution, homogeneity of variance and sphericity were verified using the Shapiro–Wilk, Levene, and Mauchly sphericity tests, respectively.

In order to normalise the impact of response rates within and between experiments, active lever presses under each dose were expressed as percentage of vehicle and used to compare the influence of aDLS dopamine receptor blockade or NAC treatment on heroin and cocaine seeking (extracted from Murray *et al*., 2012a,b, respectively) at early and late stages using two‐way anovas with dose and stage as within‐subject factors.

Significant interactions were analysed further using Newman–Keuls *post hoc* analyses. For all analyses, significance was set at α = 0.05. Effect sizes are reported as partial eta squared ($p\eta^{2}$) values (Murray *et al*., 2015).

## Results

### The control of heroin seeking behaviour progressively devolves to dorsolateral striatum dopamine‐dependent mechanisms

**Figure 2:**
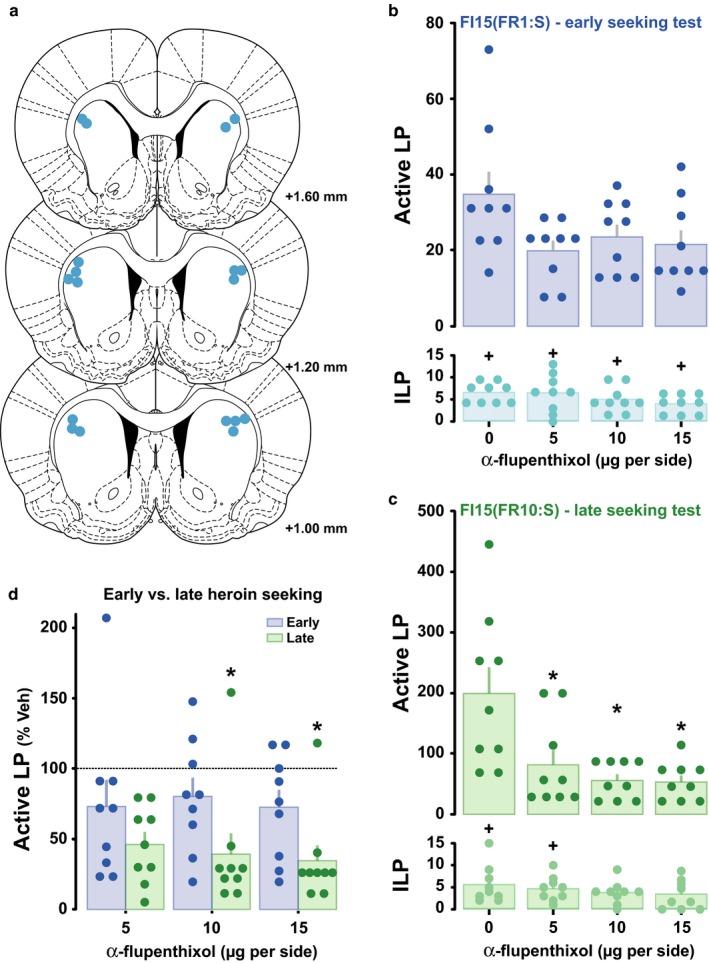
Progressive recruitment of dopamine‐dependent dorsolateral striatal control over heroin seeking. (a) Schematic representations of the localisation of dorsolateral striatal injection sites in the assessment of dopamine control of heroin seeking behaviour. Bilateral infusions of α‐flupenthixol into the anterior dorsolateral striatum had no influence on early stage heroin seeking measured during a 15‐min drug‐free period (b) but dose‐dependently decreased well‐established, cue‐controlled heroin seeking behaviour as revealed by the selective decrease in active lever presses measured during the first interval of a session under a second‐order schedule of reinforcement (c). Controlling for differential rates of responding, a clear interaction was revealed between the extent of training and the behavioural sensitivity to aDLS dopamine receptor blockade (d). *P < 0.05 vs. vehicle, ^+^ *P* < 0.05 vs. active lever presses, *n* = 9.

All animals included in the behavioural statistical analyses had cannulae located bilaterally within the targeted striatal areas as represented by the schematic representation of the location of the injector tips in the aDLS (Fig. 2a).

Once rats had acquired stable levels of heroin self‐administration under FR1 (9 ± 1 infusion/session) over 5 and 10 daily sessions, the sensitivity of early‐stage heroin seeking to DLS dopamine receptor blockade was assessed under conditions previously shown not to recruit DLS dopamine‐dependent control over cocaine seeking (Murray *et al*., 2012a, 2013, 2015). In rats with a limited history of responding under continuous reinforcement, infusions of α‐flupenthixol bilaterally into the DLS decreased only marginally (not reaching *P* < 0.05) early‐stage heroin seeking, measured over a 15‐min drug‐free period wherein every lever press resulted in a contingent presentation of the drug‐paired CS (main effect of Lever: *F*_1,8_ = 144.44, *P *<* *0.0001, $p\eta^{2}$ = 0.95, dose, *F*_3,24_ = 2.71, *P* = 0.068, $p\eta^{2}$ = 0.25, and dose × lever interaction, *F*_3,24_ = 2.02, *P* = 0.138, $p\eta^{2}$ = 0.20). Thus, active lever presses were significantly higher than inactive lever presses across the doses of α‐flupenthixol infused into the aDLS, and did not differ from those observed following vehicle infusions (Fig. 2b).

In marked contrast, the same manipulations performed after rats had been trained to seek heroin daily for prolonged periods of time under the control of heroin‐paired cues markedly decreased well‐established, cue‐controlled heroin seeking (Fig. 2c). Thus, infusions of α‐flupenthixol bilaterally into the aDLS dose‐dependently decreased active lever presses during the first interval of responding for, and prior to, the first heroin infusion (main effect of Lever: *F*_1,8_ = 28.37, *P *=* *0.00071, $p\eta^{2}$ = 0.78, dose: *F*_3,24_ = 10.01, *P *=* *0.00018, $p\eta^{2}$ = 0.56, and dose × lever interaction: *F*_3,24_ = 9.56, *P *=* *0.00025, $p\eta^{2}$ = 0.54). *Post hoc* analysis confirmed that active lever presses were decreased at all test doses compared to vehicle (*P*s < 0.05), even decreasing to inactive lever press levels at the doses of 10 and 15 μg/side. A direct comparison of performance as percentage of vehicle between early and late stages of training confirmed that only once rats had been extensively trained to seek heroin under the control of drug‐paired CSs, did instrumental responding become sensitive to aDLS dopamine receptor blockade (Fig. 2d; main effect of training stage on the inhibition of responding: *F*_1,8_ = 8.07, *P *=* *0.022, $p\eta^{2}$ = 0.5).

**Figure 3:**
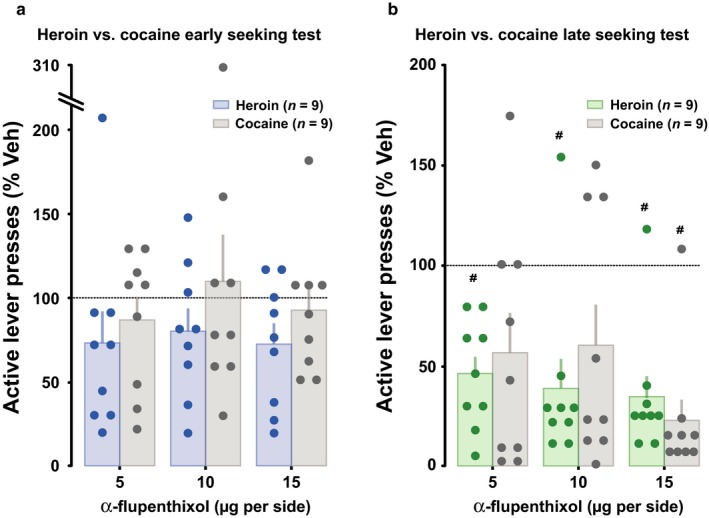
The functional recruitment of dorsolateral striatum dopamine‐dependent control over behaviour does not differ between cocaine and heroin. The magnitude of the influence of bilateral infusions of α‐flupenthixol into the anterior dorsolateral striatum on early (a), and late stage (b) heroin seeking behaviour was comparable to the one previously observed for cocaine. ^#^Indicates a significant difference from baseline lever pressing. Cocaine data are derived from (Murray *et al*., 2012a).

In order better to understand the dynamics of the progressive functional recruitment of aDLS dopamine‐dependent control over drug seeking that we have now established to occur across classes of drugs, we subsequently compared the effect of bilateral intra aDLS infusions of α‐flupenthixol on heroin (present study) and cocaine seeking (data from Murray *et al*., 2012b). Analysis of the normalised active responses of these two independent experiments revealed that the recruitment of aDLS dopamine‐dependent heroin seeking mechanisms does not differ, qualitatively or quantitatively from that previously observed for cocaine (Murray *et al*., 2012b, 2013). Thus, the effects of bilateral infusions of α‐flupenthixol into the aDLS relative to vehicle were of similar magnitude between the two drugs at both early (main effect of dose: *F*_3,48_ = 1.07, *P *=* *0.37, $p\eta^{2}$ = 0.06, drug: *F*_1,29_ = 1.29, *P *= 0.27, $p\eta^{2}$ = 0.07, and drug × dose interaction, *F*_3,48_ < 1, $p\eta^{2}$ = 0.02; Fig. 3a) and late stage training (main effect of dose *F*_3,48_ = 12.38, *P* < 0.0001, $p\eta^{2}$ = 0.43, drug: *F*_1,16_ < 1, $p\eta^{2}$ = 0.01, and dose × drug interaction: *F*_3,48_ < 1, $p\eta^{2}$ = 0.04; Fig. 3b).

### N‐acetylcysteine decreases dorsolateral striatum‐dependent heroin seeking behaviour

Having established that aDLS dopamine‐dependent mechanisms are functionally recruited when heroin seeking becomes well established, we investigated whether N‐acetylcysteine, which decreases aDLS‐dependent cue‐controlled cocaine‐seeking habits (Murray *et al*., 2012b) and nucleus accumbens‐dependent reinstatement of an extinguished instrumental response for heroin (Zhou & Kalivas, 2008) also influences cue‐controlled heroin seeking, prior to, and after the development of aDLS‐dependent responding.

**Figure 4:**
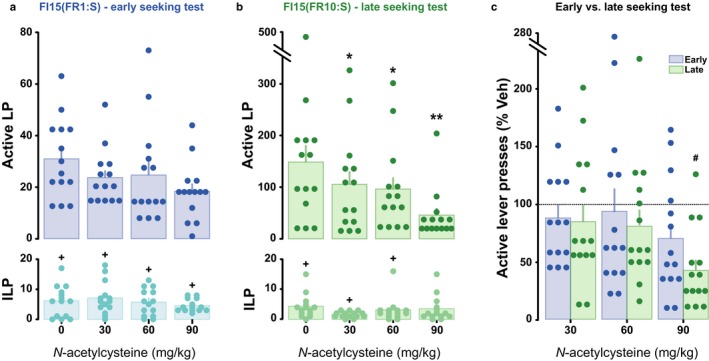
N‐acetylcysteine impairs dorsolateral striatum‐dependent cue‐controlled heroin seeking. Acute N‐acetylcysteine pretreatment did not significantly alter instrumental responding on early stage heroin seeking tests (a), but dose‐dependently decreased well‐established, cue‐controlled heroin seeking (b). The comparison of performance as percentage of vehicle between early and late stages of training showed that the influence of N‐acetylcysteine on heroin seeking behaviour was dependent on dose, but not on training history (c). *P < 0.05 vs. vehicle, ^+^ *P* < 0.05 vs. active lever presses, ^#^indicates a significant difference from baseline lever pressing, *n* = 14.

A separate cohort of rats was trained to acquire heroin self‐administration under conditions shown in this study not to recruit DLS dopamine‐dependent control over responding. After 5 to 10 daily sessions of training under FR1 schedule, rats displayed a stable rate of intake (9 ± 1 infusions/session) that was similar to the one observed in the first cohort. They were subsequently challenged with systemic IP administrations of 0, 30, 60 or 90 mg/kg NAC prior to a FI15(FR1:S) heroin seeking challenge (identical to that performed in the first experiment). At this early stage of training, NAC did not influence instrumental responding for heroin over the 15‐min drug‐free seeking sessions (Fig. 4a) even if the size of the dose effect was relatively large (main effect of dose: *F*_3,39_ = 2.26, *P *=* *0.096, $p\eta^{2}$ = 0.15, Lever: *F*_1,13_ = 48.02, *P* <* *0.0001, $p\eta^{2}$ = 0.79, and dose × lever interaction: *F*_3,39_ = 2.25, *P *=* *0.096, $p\eta^{2}$ = 0.15).

After 15 daily sessions of training to seek heroin under a second‐order schedule of reinforcement, under conditions that recruit aDLS dopamine‐dependent control over behaviour, NAC significantly decreased heroin seeking (main effect of dose: *F*_3,39_ = 5.89, *P *=* *0.002, $p\eta^{2}$ = 0.31, Lever: *F*_1,13_ = 23.79, *P *=* *0.0003, $p\eta^{2}$ = 0.65, and a dose × lever interaction: *F*_3,39_ = 5.74, *P *=* *0.002, $p\eta^{2}$ = 0.31; Fig. 4b). *Post hoc* analysis revealed that NAC was effective at reducing well‐established cue‐controlled heroin seeking at all doses tested, to the point that active lever presses were not different from inactive lever presses at the dose of 90 mg/kg (all *P*s < 0.05).

As NAC had, albeit non‐statistically significant, marginal effects on heroin seeking at the early stage of training, we investigated a full model of the effect of NAC on aDLS‐ vs. non aDLS‐dependent heroin seeking. We therefore compared the effect of NAC on normalised instrumental performance between the two stages. This analysis revealed that the effect of NAC on heroin seeking behaviour was indeed dependent on dose but not training stage (Fig. 4c), thereby suggesting that it may be effective, albeit to a lesser extent, in reducing heroin seeking also when not under aDLS control (main effect of training history: *F*_1,13_ < 1, dose: *F*_3,39_ = 6.83, *P *= 0.0008, $p\eta^{2}$ = 0.34 and training × dose interaction: *F*_3,39_ < 1).

**Figure 5:**
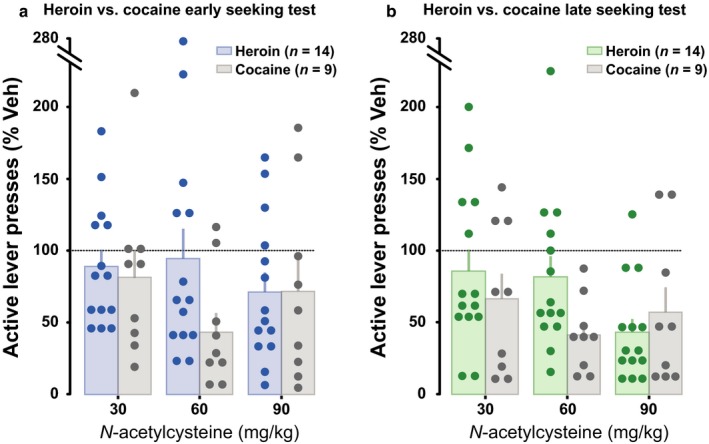
N‐acetylcysteine is equally effective at reducing cocaine and heroin seeking behaviour. Changes in active lever presses (expressed as percentage of vehicle) as a function of the dose of N‐acetylcysteine pretreatment did not significantly differ between heroin and cocaine at early (a), and late stage of drug seeking behaviour (b). Cocaine data are derived from (Murray *et al*., 2012b).

We further investigated whether the influence of NAC on heroin seeking behaviour was comparable to its effect on cocaine seeking (Murray *et al*., 2012b; Fig. 5). The comparison of the normalised seeking responses for cocaine and heroin revealed that the influence of NAC on drug seeking did not differ between heroin and cocaine at both early (Main effect of drug: *F*_1,21_ = 1.28, *P *=* *0.272, $p\eta^{2}$ = 0.06, and drug × dose interaction: *F*_3,63_ = 1.62, *P *=* *0.19, $p\eta^{2}$ = 0.08; Fig. 5a) and late stages of training (Main effect of drug: *F*_1,21_ = 1.05, *P *=* *0.316, $p\eta^{2}$ = 0.04, and drug × dose interaction: *F*_3,63_ = 2.29, *P *=* *0.086, $p\eta^{2}$ = 0.09; Fig. 5b).

## Discussion

The results of the present study demonstrate that the neural locus of control over heroin seeking behaviour progressively devolves to aDLS dopamine‐dependent mechanisms (Everitt *et al*., 2017) and that aDLS‐dependent cue‐controlled heroin seeking can be dose‐dependently decreased by N‐acetylcysteine.

Thus, in rats extensively trained instrumentally to respond for heroin infusions over protracted periods of time in a drug‐free state, but under the control of the conditioned reinforcing properties of drug‐paired cues (Everitt & Robbins, 2000, 2016), drug seeking was dose‐dependently decreased by bilateral dopamine receptor blockade in the aDLS using α‐flupenthixol microinjections which did not disrupt heroin seeking when training had been under continuous reinforcement. This is in agreement with the evidence that dopamine receptor blockade in the aDLS reduces context‐induced reinstatement of heroin seeking (Bossert *et al*., 2009) and the previous demonstration that cue‐controlled cocaine‐seeking behaviour also progressively becomes controlled by dopaminergic mechanisms in the aDLS (Vanderschuren *et al*., 2005) that are recruited by antecedent processes in the AcbC (Belin & Everitt, 2008; Willuhn *et al*., 2012).

The direct comparison of the effect of aDLS dopamine receptor blockade on early and late performance of heroin seeking to those previously described for cocaine (Murray *et al*., 2012a) revealed that the devolution of control over behaviour to aDLS dopaminergic mechanisms is quantitatively similar between the two drugs. This observation offers further experimental support to the evidence that cue‐controlled drug seeking becomes progressively reliant on the aDLS, which suggests it becomes habitual (Everitt & Robbins, 2005). Similarly, in rats trained to seek nicotine (Clemens *et al*., 2014), alcohol (Corbit *et al*., 2012), or cocaine, the latter under a seeking‐taking chained schedule of reinforcement (Zapata *et al*., 2010), drug seeking is initially goal‐directed in that is it sensitive to outcome devaluation (Adams & Dickinson, 1981; Yin *et al*., 2004, 2005; Hilario & Costa, 2008). However, after extended exposure, drug seeking becomes habitual, that is impervious to manipulation of the motivational value of the outcome of the seeking response, the behavioural signature of stimulus response, or habitual control over behaviour (Yin & Knowlton, 2006; Hilario & Costa, 2008).

At the neural systems level, this transition from goal‐directed drug seeking to habits has been shown to reflect a functional transition from dorsomedial to dorsolateral striatum in the control over foraging for cocaine and alcohol. Thus, alcohol or cocaine seeking initially relies on the posterior dorsomedial striatum (Corbit *et al*., 2012; Murray *et al*., 2012a) that mediates action‐outcome instrumental associations (Yin *et al*., 2005; Yin & Knowlton, 2006), but becomes sensitive to manipulations of, and is mediated by, the aDLS after extended training (Vanderschuren *et al*., 2005; Belin & Everitt, 2008; Zapata *et al*., 2010; Corbit *et al*., 2012). Cellular and molecular alterations in the DLS have also been observed following context‐induced reinstatement of instrumental responding for methamphetamine (Rubio *et al*., 2015), thereby suggesting that the aDLS may become progressively engaged both by cues and context after exposure to several classes of drugs. Progressive functional recruitment of cue‐elicited dorsal striatal dopaminergic mechanisms over the course of drug use history (Belin *et al*., 2013) has also been shown in humans (Volkow *et al*., 2006), even before the onset of a diagnosis of addiction (Cox *et al*., 2017), thereby suggesting that, at least in some individuals, addiction may reflect the loss of control over drug‐seeking habits.

The finding that well‐established cue‐controlled heroin seeking relies as much on aDLS dopaminergic mechanisms as cocaine‐seeking habits is in marked contrast to the differential involvement of dopaminergic mechanisms in the nucleus accumbens in mediating the reinforcing properties of cocaine and heroin. Thus, dopamine depletion in the nucleus accumbens abolishes cocaine, but not heroin self‐administration (Pettit *et al*., 1984) and the effects of dopamine and opioid receptor antagonism on cocaine and heroin self‐administration are doubly‐dissociable (Ettenberg *et al*., 1982). Consistent with the notion that under continuous reinforcement, rats titrate an optimal drug level (Wise & Bozarth, 1981), α‐flupenthixol dose‐dependently increased cocaine, but not heroin self‐administration, whereas naltrexone dose‐dependently increased heroin, but not cocaine self‐administration, indicating that direct heroin reinforcement was not reliant on dopaminergic mechanisms. In marked contrast, the present study provides evidence that dopamine receptor blockade in the aDLS disrupts heroin seeking. Therefore, the recruitment of the dorsolateral dopaminergic circuitry in maintaining *instrumental* drug *seeking* rather than *consummatory* drug *taking* appears to be independent of drug class.

This differentiation between cue‐controlled drug seeking over prolonged periods of time prior to drug availability and drug reinforcement/reward suggests that invigoration of responding brought about by conditioned reinforcers in rats engaged in well‐established cocaine or heroin seeking is mediated by a common circuitry that is distinct from that mediating reinforcement/reward mechanisms (Belin *et al*., 2013). Overall, cue‐controlled cocaine and heroin seeking both involve dopamine, GABA(B) (Di Ciano & Everitt, 2003), and μ‐opioid receptor‐dependent mechanisms (Giuliano *et al*., 2013). The rather counter‐intuitive reliance of cue‐controlled drug seeking on opioidergic mechanisms may be related to their involvement in the basolateral amygdala in mediating the motivational control of CSs over instrumental performance (Lichtenberg & Wassum, 2017).

The present study further offers evidence that N‐acetylcysteine dose‐dependently decreases aDLS dopamine‐dependent well‐established cue‐controlled heroin seeking, an effect that was quantitatively similar to that previously reported for cocaine (Murray *et al*., 2012b). While it has been previously shown that glutamate transmission in the aDLS is as important as dopamine in mediating drug seeking, but not early performance, under a second‐order schedule of reinforcement (Vanderschuren *et al*., 2005), the present data suggest that deficits in astrocyte‐controlled synaptic glutamate clearance, which is restored by N‐acetylcysteine (Moussawi *et al*., 2009), are involved in the persistence of heroin seeking, as shown previously for cocaine (Murray *et al*., 2012b) and cocaine‐induced development of habitual control over instrumental responding for natural rewards (Corbit *et al*., 2014).

This observation is consistent with evidence that, similar to cocaine (Cornish & Kalivas, 2000), glutamatergic mechanisms are engaged by short‐term exposure to heroin self‐administration (LaLumiere & Kalivas, 2008), and subsequent alteration in glutamate homeostasis in the core of the nucleus accumbens is associated with cue‐induced reinstatement of an extinguished instrumental response for both cocaine and heroin (Reichel *et al*., 2011; Shen *et al*., 2014). While N‐acetylcysteine does not influence the reinforcing properties of heroin and cocaine or the expression of escalation of cocaine self‐administration (Ducret *et al*., 2016), it prevents this cue‐induced reinstatement of responding for cocaine or heroin (Zhou & Kalivas, 2008; Moussawi *et al*., 2011) and facilitates the restoration of control over cocaine intake following punishment‐induced voluntary abstinence (Ducret *et al*., 2016). Thus, the dysregulation of glutamate homeostasis initially shown at the prelimbic cortex – nucleus accumbens core synapse to be associated with the propensity to reinstate instrumental responding for cocaine as well as heroin, potentially spreads to more dorsal territories of the striatum, eventually to encompass the dorsolateral striatum in rats extensively trained to seek cocaine or heroin under the control of the conditioned reinforcing properties of the drug‐paired cues, the reversal of which by N‐acetylcysteine impairs the expression of drug‐seeking habits. Further investigations, focusing on the differential effect of intracerebral infusions of NAC in the AcbC or the aDLS on early vs. well‐established cocaine or heroin seeking may help to identify whether the striatal locus at which NAC exerts it effects does indeed shift from the AcbC to the aDLS in parallel with the functional recruitment of aDLS dopamine‐dependent mechanisms in the control over drug seeking.

## Conclusion

Taken together, the present data offer further support for the notion that, unlike the differences that exist in the neural and cellular mechanisms mediating the direct reinforcing properties of cocaine and heroin (for review, see Badiani *et al*., 2011), those that underlie cue‐controlled drug seeking seem eventually to converge on control over behaviour by the aDLS (Belin *et al*., 2013) and rely on similar dopaminergic (Belin & Everitt, 2008) and glutamatergic (Murray *et al*., 2012b) mechanisms that are amenable to treatment by NAC.

## Conflict of interest

The authors declare no competing financial interests.

## Author contributions

DB, BJE, RH and JEM designed the experiment. JEM, MF, MP and RH carried out the experiments and acquired the data. JEM, RH, MF and DB performed the statistical analyses. RH, MF and DB produced the figures. RH, JEM, BJE and DB wrote the manuscript.

**Abbreviations**
AcbC: nucleus accumbens core
aDLS: anterior dorsolateral striatum
anova: analysis of variance
CS: conditioned stimuli
DLS: dorsolateral striatum
FI: fixed interval
FR: fixed ratio
IP: intraperitoneal
NAC: N‐acetylcysteine
PBS: Phosphate‐buffered saline
SEM: standard error of the mean
$p\eta^{2}$: partial eta squared

## Data Availability

Data are available upon request from the corresponding author.
