# Supplementary material for: Heroin seeking becomes dependent on dorsal striatal dopaminergic mechanisms and can be decreased by N‐acetylcysteine
Source: Eur J Neurosci. 2018 Mar 30;50(3):2036–44. doi: 10.1111/ejn.13894 (PMC6767855; doi:10.1111/ejn.13894)
Supplement: Supplementary file 1 [file EJN-50-2036-s001.pdf]

EJN European Journal of Neuroscience FENS Federation of European Neuroscience Societies

Heroin seeking becomes dependent on dorsal striatal dopaminergic mechanisms and can be decreased by N-acetylcysteine

Ritchy Hodebourg, Jennifer E. Murray, Maxime Fouyssac, Mickaël Puaud, Barry J. Everitt & David Belin

Review timeline:

Submission date:	06 February 2018
Editorial Decision:	26 February 2018
Revision received:	26 February 2018
Accepted:	28 February 2018

Editor: Paul Bolam

[Note: This manuscript was transferred to EJN from Biological Psychiatry, under the terms of the NPRC scheme. The reviewers who assessed this manuscript at Biological Psychiatry have not granted permission for their comments to be made public.]

1st Editorial Decision 26 February 2018

Dear David,

We have carefully looked at the previous reviews of your paper that you transferred through the NPRC system and your responses to them. We are pleased to say that we are satisfied that you have addressed the reviewers' concerns and thus do not feel that it needs to go out for additional review. Before we can accept it however, there a few issues that we noted that need to be addressed in a revised version of the manuscript.

- The use of the '%' symbol to indicate significance in Fig 3 is a little confusing since you dealing with percentages.
- Were experiments also conducted in accordance with the EU Directive? Please indicate that you had institutional ethical permission to perform the experiments.
- Please remove the 'Significance statement'.
- Indicate source and strain of the rats and the total number used.
- Include a list of abbreviations.
- If the tissue was sectioned on a cryostat then please indicate.
- Fig 2: add 'mm' to the numbers.
- Please include precise P values for those greater than .0001
- Include some detail of post-operative care of the animals in addition to the antibiotic treatment.
- The reference list should be in alphabetical order according to the name of the first author and then chronologically.
- WHO reference probably needs more detail.
- Check the reference list for journal abbreviations and use of title case.
- Check reference list for adherence to EJN guidelines.

If you are able to respond fully to the points raised, we would be pleased to receive a revision of your paper within 30 days.

Thank you for submitting your work to EJN.

Kind regards,

Paul & John
co-Editors in Chief, EJN

EJN European Journal of Neuroscience FENS Federation of European Neuroscience Societies

Authors’ Response 26 February 2018

We were delighted that you considered whe had successfully addressed all the criticisms raised by the initial four referrees and would like to thank you for your additional suggestions on the manuscript which we have now revised, taking into account each of your comments (changes displayed in red in the revised manuscript).

Here is an itemised response to your comments:

1. The use of the '%' symbol to indicate significance in Fig 3 is a little confusing since you dealing with percentages.

➔ That’s right, thank you for this. % has now been replaced by # in both figure 3 and 4.

2. Were experiments also conducted in accordance with the EU Directive? Please indicate that you had institutional ethical permission to perform the experiments

➔ Yes, stated in the revised Ms.

3. Please remove the 'Significance statement'.

➔ Done

4. Indicate source and strain of the rats and the total number used.

➔ Done as requested

5. Include a list of abbreviations.

➔ Done as requested

6. If the tissue was sectioned on a cryostat then please indicate.

➔ Yes it was, and it is now indicated.

7. Fig 2: add 'mm' to the numbers.

➔ Done as requested

8. Please include precise P values for those greater than .0001

➔ Done as requested

9. Include some detail of post-operative care of the animals in addition to the antibiotic treatment.

➔ Done as requested. Actually, since all the animals recovered perfectly from surgery no further post-op treatment was administered, but it is not explained in the revised Ms.

10. The reference list should be in alphabetical order according to the name of the first author and then chronologically.

➔ Done as requested

11. WHO reference probably needs more detail.

➔ Done, as it is a website, we now refer to the URL as well.

12. Check reference list for adherence to EJN guidelines

➔ Done as requested

Thank you very much again for your thorough editorial work on our manuscript. We hope this revised manuscript now fulfils the standards for being accepted for publication in European Journal of Neuroscience.
